# The Collagen Receptor Discoidin Domain Receptor 1b Enhances Integrin β1-Mediated Cell Migration by Interacting With Talin and Promoting Rac1 Activation

**DOI:** 10.3389/fcell.2022.836797

**Published:** 2022-03-03

**Authors:** Corina M. Borza, Gema Bolas, Xiuqi Zhang, Mary Beth Browning Monroe, Ming-Zhi Zhang, Jens Meiler, Marcin J. Skwark, Raymond C. Harris, Lynne A. Lapierre, James R. Goldenring, Magnus Hook, Jose Rivera, Kyle L. Brown, Birgit Leitinger, Matthew J. Tyska, Markus Moser, Ralph T. Böttcher, Roy Zent, Ambra Pozzi

**Affiliations:** ^1^ Department of Medicine, Division of Nephrology, Vanderbilt University, Nashville, TN, United States; ^2^ Texas A&M Health Science Center Institute of Biosciences and Technology, Houston, TX, United States; ^3^ Department of Chemistry, Vanderbilt University, Nashville, TN, United States; ^4^ Leipzig University Medical School, Institute for Drug Discovery, Leipzig, Germany; ^5^ Department of Surgery, Vanderbilt University, Nashville, TN, United States; ^6^ Veterans Affairs Hospital, Nashville, TN, United States; ^7^ Department of Cell and Developmental Biology, Vanderbilt University, Nashville, TN, United States; ^8^ National Heart and Lung Institute, Imperial College London, London, United Kingdom; ^9^ Department for Molecular Medicine, Max-Planck-Institute of Biochemistry, Martinsried, Germany

**Keywords:** receptor tyrosine kinase, integrins, extracellular matrix, migration, receptor activation, Rac1

## Abstract

Integrins and discoidin domain receptors (DDRs) 1 and 2 promote cell adhesion and migration on both fibrillar and non fibrillar collagens. Collagen I contains DDR and integrin selective binding motifs; however, the relative contribution of these two receptors in regulating cell migration is unclear. DDR1 has five isoforms (DDR1a-e), with most cells expressing the DDR1a and DDR1b isoforms. We show that human embryonic kidney 293 cells expressing DDR1b migrate more than DDR1a expressing cells on DDR selective substrata as well as on collagen I *in vitro*. In addition, DDR1b expressing cells show increased lung colonization after tail vein injection in nude mice. DDR1a and DDR1b differ from each other by an extra 37 amino acids in the DDR1b cytoplasmic domain. Interestingly, these 37 amino acids contain an NPxY motif which is a central control module within the cytoplasmic domain of β integrins and acts by binding scaffold proteins, including talin. Using purified recombinant DDR1 cytoplasmic tail proteins, we show that DDR1b directly binds talin with higher affinity than DDR1a. In cells, DDR1b, but not DDR1a, colocalizes with talin and integrin β1 to focal adhesions and enhances integrin β1-mediated cell migration. Moreover, we show that DDR1b promotes cell migration by enhancing Rac1 activation. Mechanistically DDR1b interacts with the GTPase-activating protein (GAP) Breakpoint cluster region protein (BCR) thus reducing its GAP activity and enhancing Rac activation. Our study identifies DDR1b as a major driver of cell migration and talin and BCR as key players in the interplay between integrins and DDR1b in regulating cell migration.

## Introduction

Cell adhesion and migration on fibrillar and non fibrillar collagens are mediated by several cell adhesion receptors such as integrins (Kechagia et al., 2019; Bourgot et al., 2020) and discoidin domain receptors (DDRs) (Itoh, 2018).

DDRs are receptor tyrosine kinases that consist of two closely related members DDR1 and DDR2. Upon collagen binding, DDRs undergo autophosphorylation on multiple tyrosine residues and initiate various downstream signaling pathways that regulate multiple cellular functions including cell adhesion and migration (Borza and Pozzi, 2014). DDR1 has emerged as an important contributor and a therapeutic target in cancer growth and metastasis (Rikova et al., 2007; Lee et al., 2019; Deng et al., 2021; Sun et al., 2021). To this end, DDR1 is upregulated in cancer cells and it is directly involved in matrix remodeling, tumor cell migration, invasion and metastasis (Lee et al., 2018; Chen et al., 2020; Lin et al., 2020; Romayor et al., 2021). However, the mechanism whereby DDRs mediate cell adhesion and migration and whether they promote these cell functions in an integrin-dependent and/or -independent manner is poorly understood.

Even though DDRs and integrins have non overlapping and selective collagen binding sites (Curat et al., 2001; Leitinger, 2003; Xu et al., 2011), DDRs and integrins can crosstalk with each other, thus influencing collagen-mediated cell adhesion and migration. To this end, DDR1 enhances the activation state of collagen-binding integrins α1β1 and α2β1, which strengthens their adhesion to collagen (Xu et al., 2012). Moreover, integrin α2β1 and DDR1 cooperate in regulating pancreatic cancer cells scattering (Shintani et al., 2008). In contrast to these findings, DDR1 seems to prevent collagen-induced epithelial cell migration, spreading and tubulogenesis by inhibiting integrin α2β1-mediated Cdc42 activation and STAT3 phosphorylation (Wang et al., 2006; Yeh et al., 2009). In support of a role for DDR1 in promoting cell migration, DDR1 overexpression in fibroblasts increases migration due to an interaction between DDR1 with the non-muscle myosin IIA (NMHC-IIA) (Xu et al., 2011). Interaction with NMIIA also promotes collagen fibers realignment by enhancing tractional remodeling forces (Coelho et al., 2017). Moreover, DDR1 has been shown to promote collective cancer cell invasion by coordinating the Par3/Par6 cell-polarity complex (Hidalgo-Carcedo et al., 2011); however, whether this is via an integrin dependent or independent mechanism is unclear.

DDR1 has five isoforms (DDR1a-e), with most cells expressing the DDR1a and DDR1b isoforms (Alves et al., 1995). However, the relative contribution of DDR1a and DDR1b to cell adhesion, migration, and invasion is unclear. To this end, overexpression of DDR1a, but not DDR1b, stimulates cell migration and invasion of glioma cells (Ram et al., 2006). However, in non-small cell lung carcinomas both isoforms promote cell migration upon collagen stimulation (Yang et al., 2010), while overexpression of DDR1b inhibits lung colonization of HT1080-derived tumors (Wasinski et al., 2020).

Although the mechanism underlying the functional differences between DDR1a and DDR1b is unknown, structural differences between isoforms have been suggested. Both isoforms encode functional receptor tyrosine kinases and the only difference between DDR1a and DDR1b is an extra 37 amino acids with an NPxY motif in the DDR1b intracellular juxtamembrane region preceding the kinase domain (Alves et al., 1995). Interestingly, the integrin β cytoplasmic tail contains NPxY motifs essential for recruitment of focal adhesion proteins such as talin and kindlins (Calderwood et al., 2002; Moser et al., 2009). This interaction increases integrin affinity for extracellular matrix components, thus facilitating interactions with the actin cytoskeleton and favoring cell adhesion, spreading and migration (Rognoni et al., 2016; Theodosiou et al., 2016; Sun et al., 2019).

In this study, we provide evidence that DDR1b is more effective than DDR1a in mediating integrin-dependent and -independent cell adhesion and migration. Mechanistically, DDR1b binds talin and it colocalizes with talin and integrin β1 to focal adhesions. Moreover, we show that DDR1b stimulates cell migration on collagen I by promoting the activation of the small GTPase Rac1, thus revealing a novel DDR1b-Rac1 axis.

## Results

### Increased Lung Colonization of DDR1b-Expressing Cells

**FIGURE 1:**
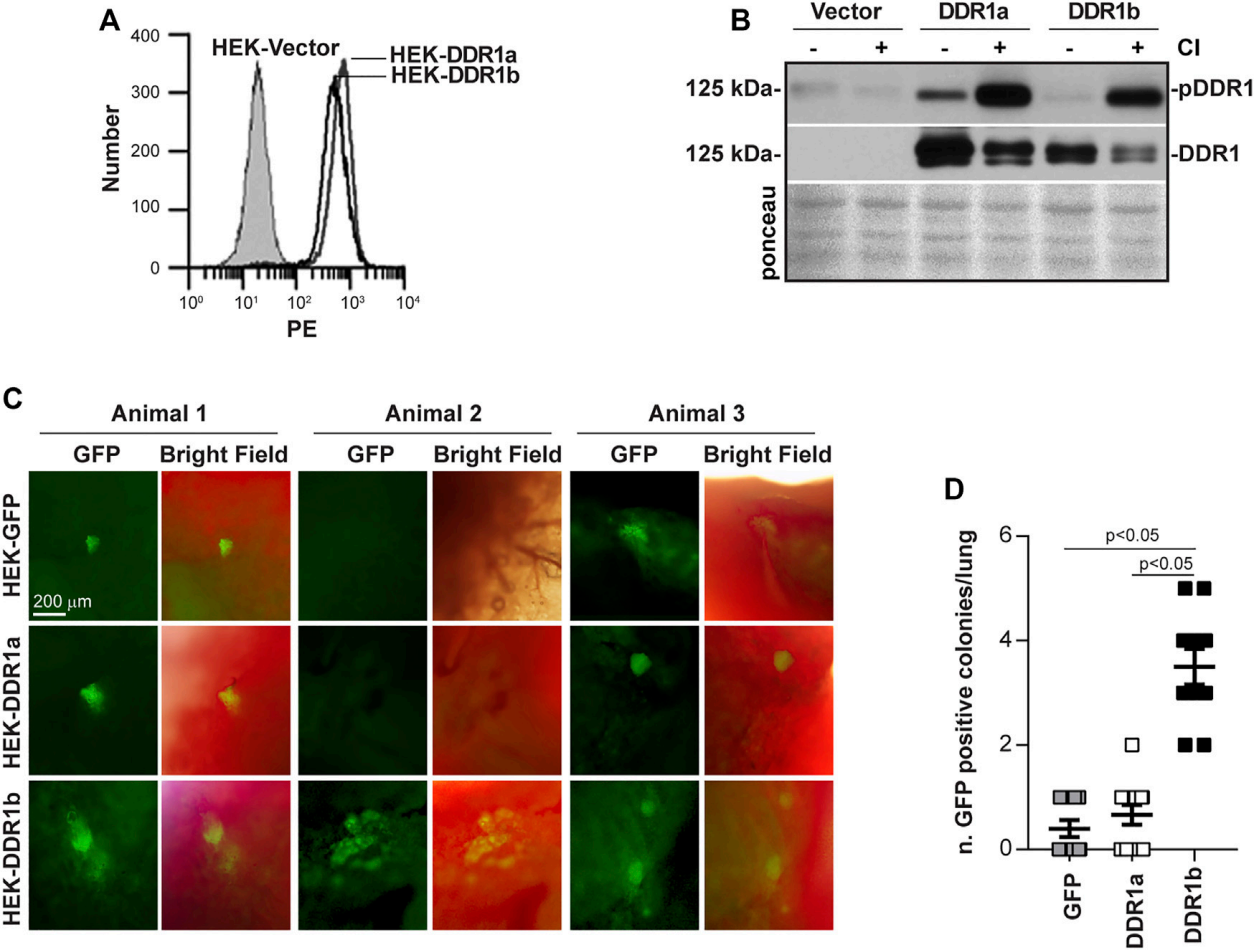
DDR1b supports lung colonization *in vivo*. **(A)** HEK cells were transfected with either empty vector, DDR1a or DDR1b cDNAs and cell populations expressing comparable levels of DDR1a and DDR1b were sorted by FACS. PE, phycoerythrin. **(B)** Western blot analysis of phosphorylated DDR1 (anti-pY792 in the activation loop of the receptor) and total DDR1 levels in HEK-Vector, HEK-DDR1a and HEK-DDR1b cells treated with acetic acid (20 mM) or collagen I (50 µg/ml in 20 mM acetic acid) for 90 min **(C)** GFP-expressing HEK-Vector, HEK-DDR1a or HEK-DDR1b cells were injected in nude mice intravenously and 2 weeks later the number of GFP positive colonies in the lungs was evaluated by placing the lung under an epifluorescence microscope. Images of lungs from 3 animals for each group are shown. **(D)** Symbols represent number of colonies per lung, whereas bars are mean ± SEM. Statistical analysis: One-way ANOVA followed by Tukey’s multiple comparison tests.

The collagen binding receptor DDR1 has been shown to contribute to cell migration (Itoh, 2018). However, the relative contribution of the two major DDR1 isoforms 1a and 1b in mediating this cell function is unclear. Since human embryonic kidney (HEK) cells are tumorigenic *in vivo* (Scherpereel et al., 2003; Macias-Perez et al., 2008), we generated stable cell lines of HEK cells expressing comparable levels of human DDR1a and DDR1b (Figure 1A) and GFP. Compared to HEK cells transfected with vector alone which express low/undetectable levels of endogenous DDR1 (Figure 1B)**,** robust DDR1a and DDR1b expression was detected by western blot in DDR1a- and DDR1b-expressing HEK cells (Figure 1B). Moreover, both DDR1a and DDR1b were phosphorylated upon treatment of cells with soluble collagen I (Figure 1B) indicating that these two receptors bind to and are activated by collagen I. We intravenously injected athymic mice with GFP-expressing HEK-Vector, HEK-DDR1a or HEK-DDR1b cells. Two weeks later, we harvested the lungs and counted the number of GFP positive colonies within the lung parenchyma. The number of colonies generated by HEK-DDR1b cells was significantly higher than those generated by HEK-Vector or HEK-DDR1a cells (Figures 1C,D), suggesting that DDR1b promotes robust cell invasion *in vivo*.

### DDR1b Promotes Adhesion and Migration on DDR Selective Substrata

**FIGURE 2:**
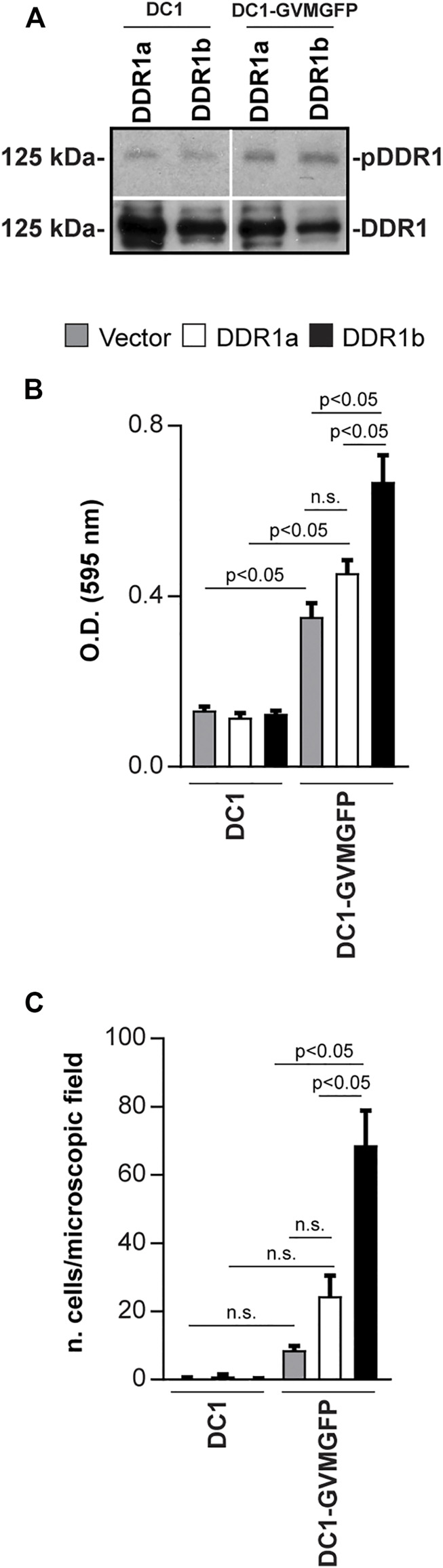
DDR1b supports cell adhesion and migration on DDR-selective substrata. **(A)** Serum-starved HEK-DDR1a or HEK-DDR1b were treated with DC1 or DC1-GVMGFP (100 µg/ml) for 90 min and the levels of phosphorylated and total DDR1 were analyzed by Western blot. **(B)** Adhesion of Vector- DDR1a- or DDR1b-expressing HEK cells on DC1 or DC1-GVMGFP constructs (30 µg/ml each). Values are the mean ± SEM of 5 independent experiments performed at least in triplicate. **(C)** Migration of Vector- DDR1a- or DDR1b-expressing HEK cells towards DC1 or DC1-GVMGFP constructs (30 μg/ml each). Values are the mean ± SEM of 3 independent experiments (for DC1-GVMGFP) with at least 6 microscopic fields counted. Statistical analysis for **(B,C):** One-way ANOVA followed by Tukey’s multiple comparison tests for pairwise comparison.

Fibrillar collagens contain specific binding sites for DDR1 (GVMGFO) and integrins α1β1 and α2β1 (GLOGEN, GFOGER) (Leitinger, 2011). To analyze the relative contribution of DDR1 in cell adhesion and migration, we generated DC1-GVMGFP, a bacterial collagen-like polypeptide containing the collagen-binding site of DDR1 in a triple helical molecule (Supplementary Figures S1A–D). These bacterial collagen-like proteins specifically bind to DDR1 and inhibit DDR1-collagen interactions (An et al., 2016). To determine whether DC1-GVMGFP activates DDR1 we treated DDR1b expressing HEK cells with different amounts of DC1-GVMGFP or DC1 (lacking the DDR1 binding site) and collagen I (CI, positive control) and then measured DDR1 phosphorylation. DC1-GVMGFP, but not DC1, induced a 3.6 ± 0.6 fold increase in DDR1 phosphorylation (three independent experiments) (Supplementary Figure S1E). We also found that in HEK-DDR1a cells, DC1-GVMGFP induced DDR1 phosphorylation, although it was to a lower extent than that detected in HEK-DDR1b cells (pDDR1/DDR1, 1.8 ± 0.6 for DDR1a vs 3.6 ± 0.6 for DDR1b) (Figure 2A).

Next, we analyzed the relative contribution of DDR1a and DDR1b to adhesion and migration on DC1 and DC1-GVMGFP. HEK-Vector, HEK-DDR1a, and HEK-DDR1b cells adhered significantly more on DC1-GVMGFP when compared to DC1 (Figure 2B). However, HEK-DDR1b cells adhered to DC1-GVMGFP significantly more than HEK-Vector and HEK-DDR1a (Figure 2B). There were no significant differences in cell adhesion between HEK-Vector and HEK-DDR1a on DC1-GVMGFP (Figure 2B) raising the possibility that endogenous DDRs in HEK cells may contribute to the adhesion on this substrate. Next, we measured cell migration and found that compared to DC1, DC1-GVMGFP promoted a significant migration only in cells expressing DDR1b, but not vector or DDR1a (Figure 2C). On DC1-GVMGFP, HEK-DDR1b cells migrated significantly more compared to HEK-Vector or HEK-DDR1a (Figure 2C). These results suggest that adhesion on and migration towards DC1-GVMGFP are integrin-independent and mediated prevalently by DDR1b.

**FIGURE 3:**
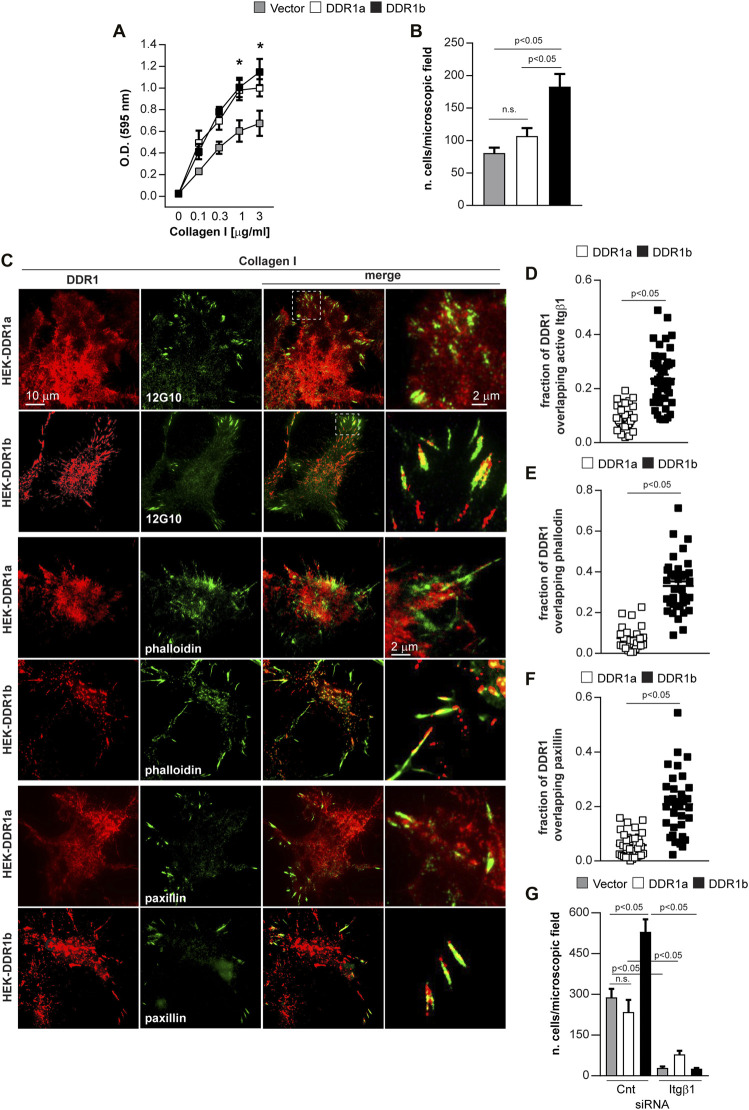
DDR1b enhances cell migration on collagen I and localizes to focal adhesions. **(A)** Adhesion of Vector- DDR1a-, or DDR1b-expressing HEK cells to collagen I (0–3 µg/ml). Values are the mean ± SEM of 3 independent experiments performed at least in triplicate. **p < 0.05 relative to HEK-Vector. **(B)** Migration of Vector- DDR1a-, or DDR1b-expressing HEK cells towards collagen I (20 μg/ml). Values are the mean ± SEM of 3 independent experiments with at least 4-6 fields/microscopic field counted. Statistical analysis in **(A,B):** One-way ANOVA followed by Tukey’s multiple comparison test. **(C)** Total internal reflection fluorescence (TIRF) microscopy of HEK-DDR1a and HEK-DDR1b cells plated on collagen I (20 µg/ml) for 1 h and then co-stained with anti-DDR1 and integrin beta 1 (12G10), FITC-phalloidin, or anti-paxillin antibodies. Overlay images reveal co-localization of DDR1b with activated integrin β1, actin or paxillin. Co-localization of DDR1a, DDR1b with 12G10 **(D)**, actin **(E)**, paxillin **(F)** at the cell membrane of cells plated on collagen I was determined using the ImageJ/JACoP analysis and the Manders’ overlap coefficient (Bolte and Cordelieres, 2006). Values are the mean ± SEM of 3-4 FAs calculated for 10-13 cells and represent the fraction of DDR1 signal overlapping with 12G10 phalloidin and paxillin. Statistical analysis: unpaired two-tailed *t*-test. **(G)** Migration of Vector- DDR1a-, and DDR1b-expressing HEK cells transfected with Cnt or Itgβ1 siRNA towards collagen I (20 μg/ml). Values are the mean ± SEM of 3 independent experiments with at least 7 fields/microscopic field counted. Statistical analysis was done with One-way ANOVA followed by Tukey’s multiple comparison test.

Since collagen I is the major ligand for DDR1 (Shrivastava et al., 1997; Vogel et al., 1997), we examined whether DDR1a and DDR1b differentially support adhesion and migration on this substrate. Compared to HEK-Vector cells, both HEK-DDR1b and HEK-DDR1a cells adhered more on collagen I, but the increase in adhesion was significant only for HEK-DDR1b cells (Figure 3A). There were no significant differences in adhesion to collagen I between HEK-DDR1a and HEK-DDR1b cells (Figure 3A). In contrast, HEK-DDR1b migrated towards collagen I significantly more than HEK-DDR1a or HEK-Vector cells (Figure 3B).

In cells plated on collagen I, DDR1b, but not DDR1a, co-localized with activated integrin β1, as determined by staining with an antibody thar recognizes the active conformation of integrin β1 (12G10) (Mould et al., 1995) (Figures 3C,D) as well as F-actin and paxillin (Figures 3C,E,F). In contrast, when cells were plated on fibronectin, we failed to detect DDR1a or DDR1b colocalization to paxillin (Supplementary Figure S2). This result indicates that DDR1b is recruited to focal adhesions (FAs), integrin-containing multi-protein structures that connect the ECM to the actomyosin cytoskeleton and transmit traction forces required for cell migration, only in the presence of collagen (Austen et al., 2015; Case and Waterman, 2015). The differences in migration between HEK-DDR1b and HEK-Vector or HEK-DDR1a were not due to increased integrin expression as the integrin β1 levels were similar in all cell types (Supplementary Figure S3A), consistent with previous reports (Xu et al., 2012).

To determine whether the effect of DDR1b on cell migration on collagen involves integrins, we depleted integrin β1 by siRNA in Vector-, DDR1a- and DDR1b-expressing HEK cells (Supplementary Figure S3B). In the control siRNA group, as expected, DDR1b significantly increased cell migration compared to Vector- or DDR1a-expressing cells (Figure 3G). Interestingly, depletion of integrin β1 equally abolished cell migration on collagen I in Vector-, DDR1a- and DDR1b-expressing cells (Figure 3G) suggesting that the effect of DDR1b on cell migration required integrin expression. Overall, on DC1-GVMGFP DDR1b mediates cell adhesion and migration in an integrin-independent manner. On collagen I, DDR1b co-localizes with activated integrin β1 to FAs and enhances integrin β1-mediated cell migration.

### DDR1b Interacts With Talin

Talin is a major component of FAs and interacts with integrin β subunits (Critchley, 2009). Moreover, talin was identified as putative DDR1-binding protein in a proteomic study in which DDR1 specific phosphopeptides were used to identify DDR1 interacting proteins (Lemeer et al., 2012). Several tyrosine residues in the DDR1 cytoplasmic tail, including Y513 within the NPxY motif of DDR1b, were identified as potential binding sites for talin and other FERM-domain-contain proteins, such as ezrin (Supplementary Figure S4A). NPxY motifs in integrins are critical for the interaction with FERM domains-containing proteins. For instance, the membrane proximal NPxY motif in the integrin β1 subunit is required for binding to talin which leads to integrin activation and regulation of cell function, including cell adhesion and migration (Moser et al., 2009; Zhu et al., 2021). Thus, we addressed whether the extra 37 amino acids encompassing the NPxY motif in DDR1b interacts directly with talin.

**FIGURE 4:**
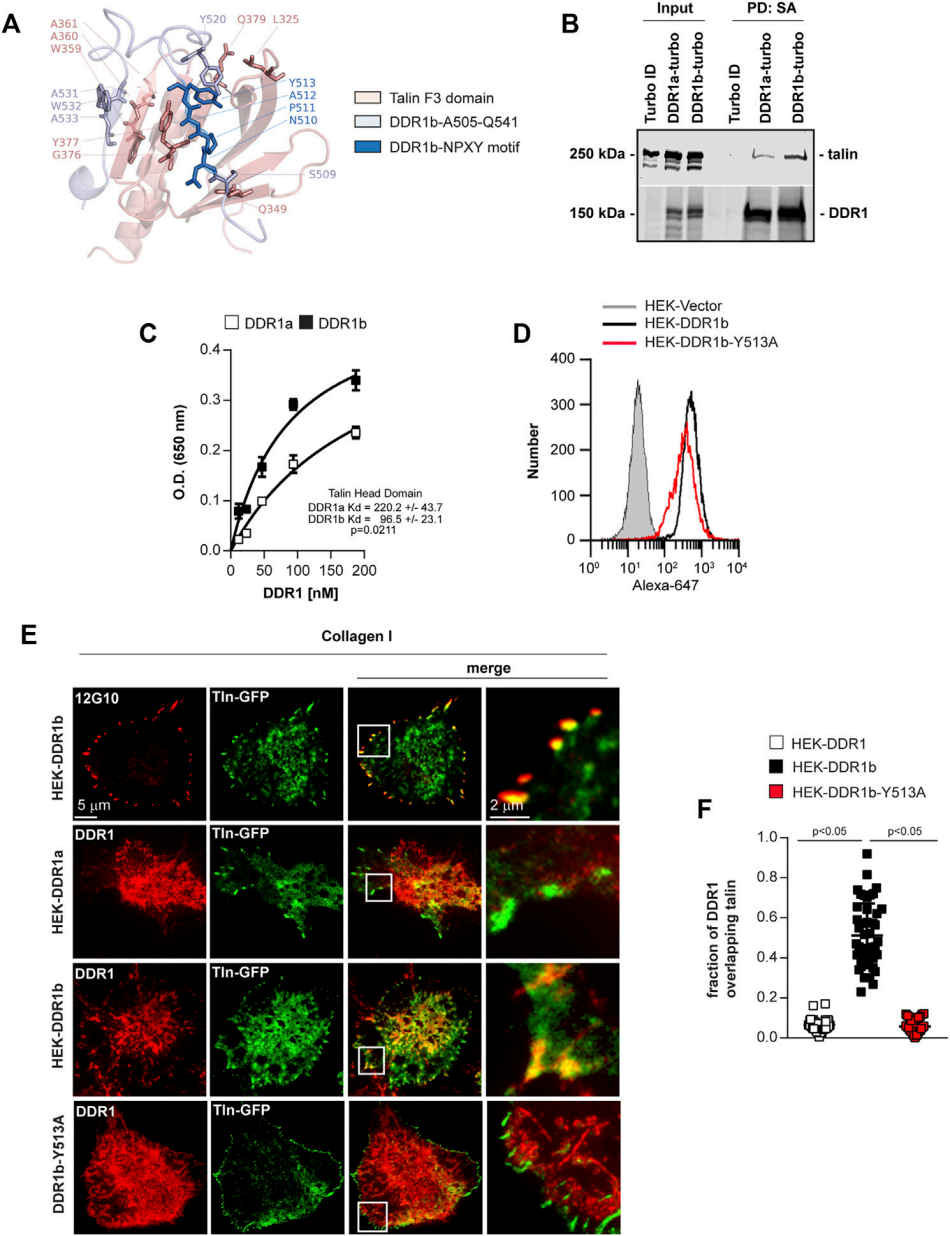
Talin binds better to DDR1b than DDR1a. **(A)** Model of binding between DDR1b intracellular domain (A505-Q541) and talin F3 domain. Talin F3 domain is in pink, DDR1b A505-Q541 is in light blue and the NPxY motif of DDR1b is highlighted in dark blue. Side chains of the amino acids discussed in text have been shown in sticks. **(B)** TurboID, DDR1a-turbo or DDR1b-turbo HEK expressing cells were plated on collagen I (30 μg/ml) for 1–3 h and then lysed and subjected to Streptavidin (SA) pull-down (PD) followed by western blotting with talin or DDR1 antibodies. **(C)** Immobilized talin head domain (3 µg/ml) was incubated with increasing amounts of purified cytoplasmic domains of DDR1a or DDR1b and the bound DDR1 was detected with anti-DDR1 antibody. Raw data in **C** were fitted with Non Linear Regression–Global Curve Fitting with one site saturation using Sigma Plot and the calculated Kd were compared using a two-tailed *t*-test. **(D)** HEK cells were stable transfected with either empty vector, DDR1b or HEK-DDR1b-Y513A cDNAs and cell populations expressing comparable levels of DDR1 were sorted by FACS. **(E)** Total internal reflection fluorescence (TIRF) microscopy of HEK-DDR1a, HEK-DDR1b and DDR1b-Y513A cells stable transfected with full length talin1-GFP plated on collagen I (20 µg/ml) for 1 h and then stained with anti-DDR1 or 12G10 antibody. Overlay images reveal co-localization of 12G10 and talin (positive control) and DDR1b with talin. **(F)** Co-localization of DDR1a, DDR1b and DDR1b-Y513A with talin1-GFP at the cell membrane of cells plated on collagen I was determined using the ImageJ/JACoP analysis and the Manders’ overlap coefficient (Bolte and Cordelieres, 2006). Values are the mean ± SEM of 3-4 FAs analyzed in more than 10 cells and represent the fraction of DDR1 signal overlapping with talin-GFP signal. Statistical analysis: one-way ANOVA followed by Tukey’s multiple comparisons test.

Based on a structure of the talin F3 domain bound to the β3 integrin tail (PDB ID: 2h7e (Wegener et al., 2007)), we constructed 600,000 tentative structures of the DDR1b juxtamembrane region docked to talin F3 domain using the Rosetta protein modeling suite (see Methods) and found that the folding/docking trajectories converged on a well-defined, low-energy cluster of models, whose lowest energy representative is illustrated in Figure 4A. According to the Rosetta energy function, residues depicted as a “stick” (Figure 4A) contribute the most to the interaction energy. In this model the DDR1b-Y513 favorably interacts with talin-Y377 and forms a hydrogen bond with talin-L325. Furthermore, Y513 also undergoes a polar contact with DDR1-Y520, which in turn makes a hydrogen bond with L325. The NPxY motif of DDR1b consisting of N510-P511-(A512)-Y513 is instrumental in maintaining the interaction with talin by forming a network of hydrogen bonds to Y377 and immediately precedes talin-G376. The W359-A360-A361 residues of talin form an antiparallel beta sheet by means of a hydrogen bond network with the A531-W532-A533 residues of DDR1, which probably maintains the same conformation present in 2h7e integrin-talin structure. Notably, in this model, talin-Q381 lacks a strong interaction with DDR1b. If confirmed, this finding is in stark contrast to the published integrin structure [PDB ID: 2h7e (Wegener et al., 2007)]. We also observed that the conformation of the C-terminal part of DDR1b linker resembles the structure of the integrin template, while the N-terminal part is clearly different. *In silico* mutagenesis analysis performed to determine the influence of amino acid substitution on computed DDR1b (A505-Q541)-talin F3 domain binding energy, confirmed a key role of DDR1b-Y513 in maintaining the interaction with talin and Q349, G376 and Y377 of talin in maintaining the interaction with DDR1b (Supplementary Figure S5). Overall, these results suggest that DDR1b will bind with higher affinity to the talin head domain (THD) than DDR1a with the NPxY motif in DDR1b being critical for the interaction.

To determine whether DDR1 co-localizes with talin in live cells, first we used a proximity-dependent labeling method, BioID. In BioID one of the proteins, the bait, is fused to a biotin ligase, turbo-Flag, which will biotinylate proteins in close proximity (∼10 nm) (Varnaite and MacNeill, 2016) to the bait. This method allows biochemical identification of transient, low affinity interactors in live cells. We generated HEK cells expressing DDR1a-turbo, DDR1b-turbo or the biotin ligase alone and plated cells on collagen I. Next, we isolated the biotinylated proteins and performed Western blot analysis with an anti-talin antibody. We found that in DDR1b-turbo cells there was a 2.29 ± 0.79 fold increase (three independent experiments) in the levels of biotinylated talin than in cells expressing DDR1a-turbo (Figure 4B), suggesting that DDR1b is in closer proximity with talin than DDR1a.

To determine whether DDR1 interacts directly with talin and to confirm the model that DDR1b binds talin with higher affinity than DDR1a, we used binding assays with purified recombinant proteins. For the binding assay, we incubated baculovirus expressed DDR1a and DDR1b cytoplasmic domains (Supplementary Figure S4A, Figure 4B) with bacterially expressed talin head domain (THD, a. a. 1–433) (Supplementary Figure S4C). For detection of bound DDR1 we used an antibody recognizing a common epitope at the C-terminus of DDR1 (Supplementary Figure S4D). We found that DDR1b bound with a significantly higher affinity than DDR1a to immobilized THD (Figure 4C).

Finally, to support the interaction of talin and DDR1b in HEK cells and to evaluate the role of NPxY motif in the interaction with talin, we expressed GFP-talin in HEK-DDR1a and HEK-DDR1b cells as well as in cells expressing a Y-to-A mutation within the NPxY motif of DDR1b (HEK-DDR1b-Y513A) (Figure 4D). Next, we examined talin co-localization with DDR1 on cells plated on collagen I. DDR1b, but not DDR1a or DDR1b-Y513A, co-localized with talin (Figures 4E,F) to FAs, thus confirming that DDR1b interacts and co-localizes with talin more than DDR1a and that the NPxY motif is critical for co-localization with talin.

### DDR1b Promotes Migration by Increasing Rac Activation

Depletion of integrin β1 abolishes DDR1b increased migration on collagen I (Figure 3F) which suggests that DDR1b enhances integrin β1-mediated cell migration. Because the closely related DDR2 was shown to activate integrins via Rap 1 mediated talin recruitment (Bayer et al., 2019), we examined Rap1 activation in DDR1a- and DDR1b-expressing cells plated on collagen I. We failed to detect a significant increase in Rap1 activation between DDR1a-vs DDR1b-expressing cells plated on collagen I (data not shown), suggesting that an alternative mechanism is responsible for the DDR1b-mediated cell migration.

**FIGURE 5:**
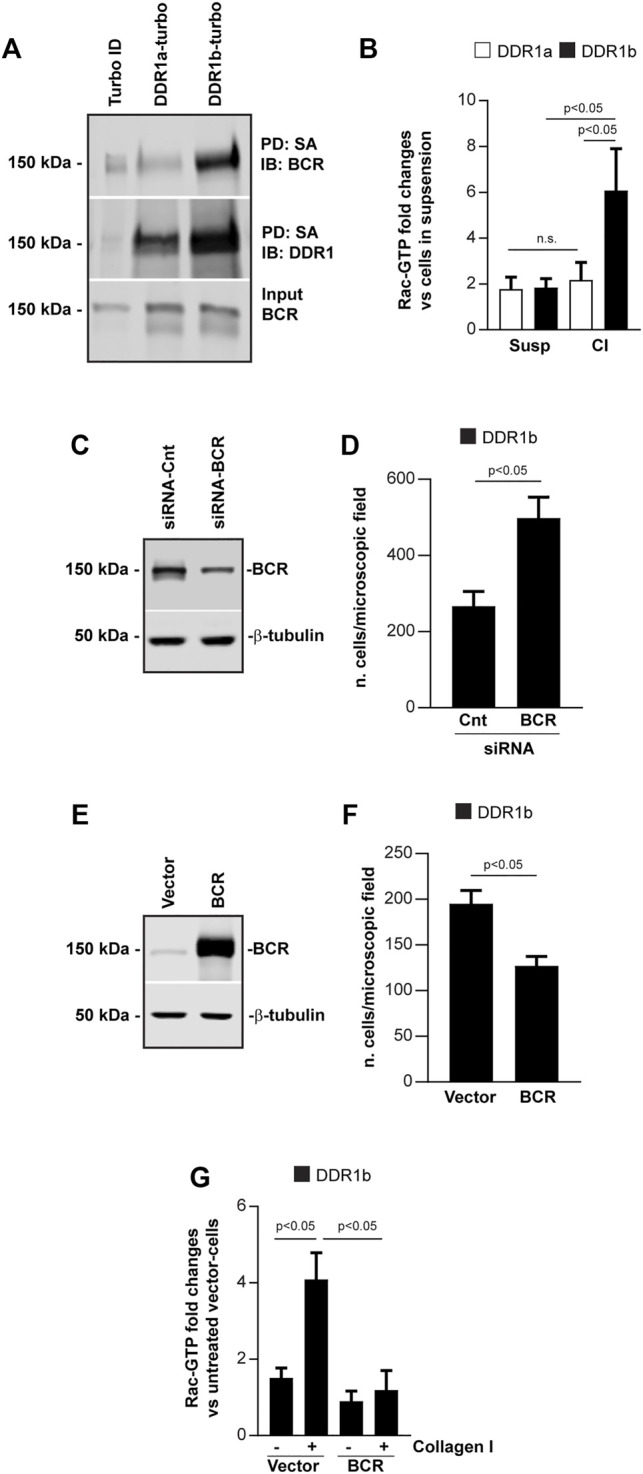
DDR1b increases cell migration by inhibiting BCR GAP activity. **(A)** Turbo, DDR1a-turbo or DDR1b-turbo HEK expressing cells plated on collagen I (30 μg/ml) for 1–3 h were lysed and subjected to streptavidin (SA) pulldown (PD) followed by Western blot (IB) with anti-BCR or anti-DDR1 antibodies. **(B)** DDR1a or DDR1b expressing cells left in suspension or plated on collagen I (30 μg/ml) for 30 min, were lysed and analyzed for the levels of active Rac. The levels of Rac-GTP were adjusted to total Rac determined by western blot analysis. Values represent fold increase relative to cells left in suspension with the lowest levels of active Rac assigned a value of 1. Values are mean ± SEM of 3 independent experiments. Statistical analysis: one-way ANOVA followed by Tukey’s multiple comparison test. **(C)** Western blot analysis of cells transfected with Cnt siRNA or BCR siRNA. One representative experiment of 3 independent experiments is shown. **(D)** Migration of DDR1b cells transfected with Cnt siRNA or BCR siRNA toward collagen I (20 μg/ml). Values are the mean ± SEM of 3 independent experiments with 7 fields/microscopic field counted**. (E)** Western blot analysis of cells transfected with BCR or empty vector. One representative experiment of 3 independent experiments is shown. **(F)** Migration of DDR1b cell transfected with BCR or empty vector towards collagen I (20 μg/ml). Values are the mean ± SEM of 3 independent experiments with 7 fields/microscopic field counted. Statistical analysis for **(D,F):** unpaired two-tail *t-test.* **(G)** DDR1b cells transfected with BCR or empty vector were left in suspension or plated on collagen I for 30 min, lysed and then analyzed for the levels of active Rac using a G-LISA assay as described above. Values represent fold increase relative to cells transfected with empty vector with the lowest levels of active Rac left in suspension assigned a value of 1. Values are mean ± SEM of 3 independent experiments. Statistical analysis: one-way ANOVA followed by Tukey’s multiple comparison test.

Breakpoint cluster region protein (BCR) is an interactor and downstream target of DDR1 (Jeitany et al., 2018) and acts as a GTPase activating protein (GAP) (Cho et al., 2007) thus accelerating deactivation of small GTPases by enhancing GTP hydrolysis. BCR regulates migration by negatively regulating the activation of the Rho GTPase Rac (Cho et al., 2007; Cunnick et al., 2009). We therefore investigated whether the increased migration of DDR1b-vs. DDR1a-expressing cells was due to DDR1b interacting with BCR thus attenuating its GAP activity towards Rac1. To do this, first we examined whether BCR is in closer proximity with DDR1b than with DDR1a using BioID. We found a 2.92 ± 1.42 fold increase (three independent experiments) in biotinylated BCR in DDR1b-expressing cells compared to DDR1a-expressing cells (Figure 5A), suggesting that BCR co-localizes with DDR1b more than with DDR1a. Next, we investigated whether DDR1a and DDR1b differentially regulate the activation of Rac1. Using a G-Lisa assay, we found that when plated on collagen I, DDR1b-expressing cells had significantly higher levels of activated Rac1-GTP compared to DDR1-a cells (Figure 5B).

Finally, to better define the role of the DDR1/BCR axis in regulating cell migration, we depleted or overexpressed BCR in DDR1b-expressing cells (as these cells have higher levels of Rac1-GTP, Figure 5B) and analyzed their migration on collagen I. We found that BCR depletion increased cell migration (Figures 5C,D) while BCR overexpression decreased cell migration (Figures 5E,F). Importantly, BCR overexpression significantly reduced Rac1 activation in DDR1b-expressing cells plated on collagen I (Figure 5G), suggesting that DDR1b potentiates Rac-1 activation by preventing BCR-mediated GAP activity.

## Discussion

**FIGURE 6:**
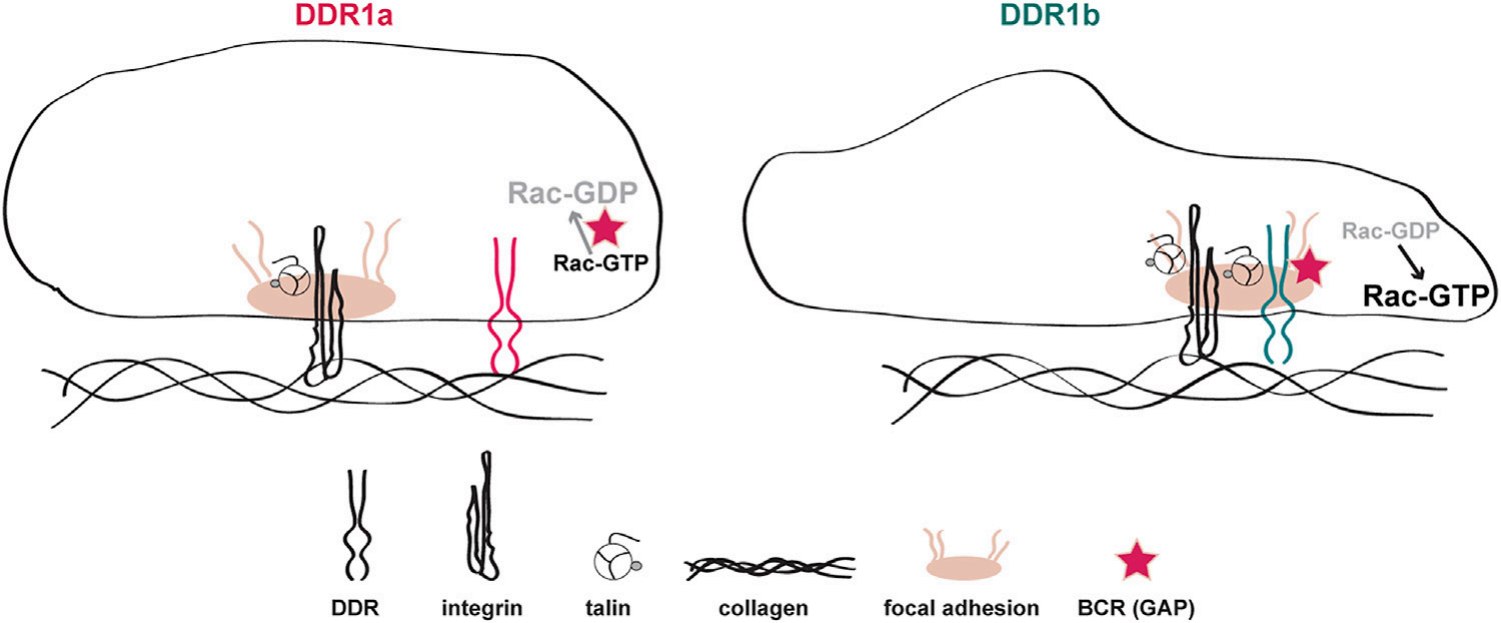
Schematic representation of collagen I-mediated DDR1a vs DDR1b function. In cells expressing DDR1a and exposed to collagen I, this receptor does not co-localize with activated integrins in focal adhesions. In contrast, in cells expressing DDR1b and exposed to collagen I, this receptor binds more efficiently to talin, co-localizes with activated integrins in focal adhesions, and enhances integrin β1-mediated cell migration. In addition, DDR1b binds the GAP BCR thus enhancing Rac1 activation and migration.

The goal of this study was to determine the relative contribution of DDR1a and DDR1b in mediating cell adhesion and migration. We provide evidence that DDR1b regulates cell adhesion, migration and invasion in an integrin-dependent and -independent manner. We show that DDR1b directly interacts with the cytoskeleton adaptor protein talin and co-localizes with talin and integrins to focal adhesions. Moreover, we show that DDR1b negatively regulates BCR GAP activity thus promoting Rac activation and in turn migration Figure 6.

Our results showing that bacterial collagen containing a selective DDR1 binding site promotes DDR1 phosphorylation differ from those reported by An et al. (2016) where no phosphorylation was observed when using their own-generated bacterial collagen. This discrepancy can be due to the fact that different bacterial collagens and/or different conditions for the receptor activation assay were used. Nevertheless, the bacterial collagens used by An et al. (2016) bind DDR1 and affect DDR1-expressing cell migration, which suggests that bacterial collagens containing DDR1 binding site are a useful tool for addressing the role of DDRs in cell migration.

Our results indicate that on the DDR1 selective substrate GVMGFP, DDR1b, but not DDR1a, promotes cell adhesion and migration. This finding is consistent with previous results indicating that DDRs can mediate integrin-independent cell adhesion (Xu et al., 2012). The extracellular domains of DDR1a and DDR1b are identical, and we generated HEK cells expressing comparable cell surface levels of these two isoforms. Thus, the differences in cell adhesion and migration exerted by DDR1b vs DDR1a are likely due to selective DDR1b-mediated recruitment of proteins controlling cell adhesion and migration, rather than differential binding to GVMGFP between the two isoforms. Moreover, we show that on collagen I, DDR1b but not DDR1a, promotes cell migration. This finding seems to disagree with previous studies showing that glioblastoma cells expressing DDR1a migrate and invade more compared to cells expressing DDR1b (Ram et al., 2006; Yang et al., 2010). This discrepancy may be attributed to cell type dependent effects or to the fact that expression of these two isoforms might alter the surface expression of integrins in tumor cells. To this end, in contrast to our study where all the cells we used express similar levels of integrin β1, integrin levels in DDR1 expressing glioblastoma cells were not investigated.

Simultaneous engagement of DDR1 and integrins via combinations of integrin- and DDR1-selective peptides was shown to synergistically increase cell adhesion compared to cells plated on integrin- or DDR1-specific peptides alone (Xu et al., 2012). Collagens contain both DDR1 and integrin binding sites and we found that depletion of integrin β1 in DDR1b-expressing cells reduces migration on collagen I to levels observed in vector-expressing cells. This result suggests that on collagen substrata DDR1b potentiates integrin-mediated cell migration rather than promoting migration in an integrin-independent manner. Several mechanisms have been proposed for DDR-mediated integrin activation. In cancer associated fibroblasts, DDR2 was shown to activate Rap1 which increases talin recruitment to integrin complexes and in turn increases integrin β1 activation (Bayer et al., 2019). DDR1 overexpression was also shown to increase integrin expression in human gingival fibroblasts due to altered glycosylation and cleavage of the integrin β1 subunit (Staudinger et al., 2013). However, neither of these mechanisms explain the increased DDR1b-mediated cell migration as the levels of integrin β1 as well as Rap1 activation are similar in DDR1a vs. DDR1b expressing cells.

In this study we show that DDR1 interacts and co-localizes with talin to FAs. Talin is essential for integrin activation, for linking the cytoplasmic tail of the integrins to the actin cytoskeleton and for stabilizing FAs. Talin consists of two major domains, the head and the rod domains. The N-terminal THD is a FERM domain consisting of F0, F1, F2, F3 subdomains. The F3 subdomain contains a phosphotyrosine-binding domain shown to bind the NPxY motif in the integrin β tails. In addition, this domain binds the hyaluronan receptor layilin, the phosphatidylinositol 4-phosphate 5-kinase type Iγ, focal adhesion kinase and the guanine nucleotide exchange factor TIAM1 (Calderwood et al., 2013). Our study shows that DDR1 interacts directly with talin and that this interaction is isoform dependent. To this end, we show that the cytoplasmic domain of DDR1b binds to talin with higher affinity than DDR1a; and DDR1b, but not DDR1a, co-localizes with talin in FAs. Both isoforms encode functional receptor tyrosine kinases with the only difference the extra 37 amino acids encompassing the NPxY motif (Alves et al., 1995). Our modeling data indicate that the NPxY motif plays a critical role in the interaction with talin. This was confirmed by mutating the Y513 in the NPxY motif of DDR1b which results in loss of localization of the receptor to FAs. Our data that DDR1b localizes to FAs differ from previous reports (Vogel et al., 2000; Coelho et al., 2017; Yeung et al., 2019). This discrepancy could be due to several factors, including the cell type used and/or the experimental conditions. For instance, the study by Vogel et al. used cells lacking integrin β1 kept in culture for 72 h prior analysis. Yeung et al. used mesenchymal cells grown on slides and then treated with soluble collagen I, a condition used to activate DDR1, but not integrins. Finally, Coelho et al. analyzed FAs at later time points (4–24 h) than the ones analyzed in this study (1 h). Overall, we show that DDR1b, but not DDR1a or DDR1bY-513A, colocalizes with talin and integrin β1 at FAs, indicating that the NPxY motif is likely to be critical for DDR1b localization to FA and for enhanced migration of DDR1b expressing cells.

Here, we show that DDR1b isoform promotes Rac1 activation by modulating BCR GAP activity. To this end, DDR1b-expresing cells have higher levels of Rac1-GTP than DDR1a-expressing cells; and BCR overexpression decreases Rac1 activation and in turn cell migration. This result is consistent with the finding that BCR inhibits macrophage directed migration by functioning as a GAP for Rac1 (Cunnick et al., 2009). Moreover, BCR was shown to regulate additional Rac1-dependent processes such as reactive oxygen species production in neutrophils (Voncken et al., 1995) and dendritic arborization in hippocampal neurons (Park et al., 2012). Whether DDR1-BCR interaction affects additional Rac1-dependent processes remains to be determined.

Interestingly, DDR1 seems to both activate and inhibit Rho GTPases in a cell type-dependent manner. In breast cancer cells, DDR1 increases Cdc42 activation and its specific guanine nucleotide-exchange factor (GEF) Tuba. This process is critical for linear invadosome formation (Juin et al., 2014). In contrast, in Madi-Darby canine kidney cells, both DDR1a and DDR1b isoform decrease Cdc42 activation and inhibit integrin-mediated cell spreading (Yeh et al., 2009). Our results clearly indicate that, in HEK293 cells, DDR1 promotes Rac1 activation and migration and that BCR is critical for this function.

How DDR1b regulates BCR GAP activity is not clear. DDR1-mediated phosphorylation of BCR inhibits BCR-β-catenin interaction thus removing the BCR inhibitory effect on β-catenin transcriptional activity (Jeitany et al., 2018). DDR1 may inhibit BCR-GAP activity by phosphorylating BCR. In this context, BCR GAP activity can be regulated by an intramolecular interaction between the N- and C-termini of BCR which is dependent on tyrosine phosphorylation (Park et al., 2012). Alternatively, BCR GAP activity can be inhibited by protein-protein interaction. This was shown for Rho GDIα which binds to the BCR GAP domain and blocks BCR GAP activity (Kweon et al., 2008). Whether DDR1b prevents BCR GAP activity via phosphorylation or *via* direct protein-protein interaction remains to be determined.

DDR1 influences cell migration through multiple mechanisms (Huang et al., 2009; Juin et al., 2014). For instance, it promotes collective cell migration independent of binding to collagen (Hidalgo-Carcedo et al., 2011) while collagen-induced DDR1 signaling is required for bladder tumor cells colonization of the lung (Lee et al., 2019). Moreover, DDR1 can influence cell migration by regulating the expression and secretion of matrix metalloproteases (Yang et al., 2010; Roberts et al., 2011). Although DDR1a and/or DDR1b might influence cell adhesion and migration through multiple mechanisms, our *in vivo* studies show that only DDR1b expressing HEK cells forms significantly more lung colonies than DDR1a or vector expressing HEK cells. Our result not only indicates that DDR1b is a major driver of cell migration and invasion, but also seems to agree with the finding that DDR1b is the isoform expressed and activated in various types of cancer, including lung and renal cancers (Ambrogio et al., 2016; Haake et al., 2016).

In conclusion, our study identifies DDR1b as a major driver of cell adhesion, migration, and invasion in an integrin-dependent and -independent manner. DDR1b promotes migration by interacting and colocalizing with talin and integrins to FAs and by promoting Rac activation through inhibition of BCR GAP activity.

## Materials and Methods

### Plasmids

To generate full length pIRES-DDR1a the cDNA was released from pRK5-DDR1a with *EcoRI* and *BamHI* and cloned between the same site in pIRES-puro. The generation of pIRES-DDR1b and DDR1b-Turbo containing a Flag tag was previously described (Borza et al., 2017; Borza et al., 2021). DDR1a-turbo and pIRES-DDR1b-Y513A were generated with QuikChange II XL Site-Directed Mutagenesis Kit (Agilent Technologies) using the primers 5′-CCA​CTG​TAG​GCA​GAG​CCA​TTG​GGG​ACA​CAG​G-3′ and 5′-CCT​GTG​TCC​CCA​ATG​GCT​CTG​CCT​ACA​GTG​G-3′ for DDR1a and 5′-GCC​AGA​AGG​AGG​CGG​GCG​GCT​GGA​TTG​GAA​GAG-3′ and 5′-GCC​AGA​AGG​AGG​CGG​GCG​GCT​GGA​TTG​GAG​AG-3′ for DDR1b-Y513A following the manufacturer’s instructions. GFP-Talin containing full length mouse talin-1 cDNA was a gift from S.J. Monkley (Praekelt et al., 2012), talin head domain HIS-tagged (THD, a. a. 1–433) was a gift from M. Ginsberg (Kim et al., 2012), murine BCR-Flag tagged cDNA was previously described (Borza et al., 2021). To generate DC1-GVMGFP, the Scl2.28 DNA sequence from *S. pyogenes* was optimized for expression in *E. coli* and ligated into the *E. coli* vector, pColi (Cosgriff-Hernandez et al., 2010; Seo et al., 2010). A DNA segment encoding the DDR1 binding motif from collagen I (GVMGFP) was introduced into pScl2.28 by overlap extension PCR using primers from Integrated DNA Technologies. The insertion (position 163 Pro → position 166 Leu replaced with VMGF to generate DC1-GVMGFP) was verified by sequencing.

### Antibodies

The following antibodies were used: DDR1 (Santa Cruz SC-532, 1:4,000 for WB and 1:2000 for ELISA; Cell Signaling #5583, clone D1G6, 1:2000 for WB and 1:1,000 for IF; mAb 7A9 (Carafoli et al., 2012), 1:200 for FACS; R&D AF2396, 1:50 for FACS and 1:2000 for WB); pY792-DDR1 (Cell Signaling #11994, 1:1,000 for WB); Paxillin (BD Biosciences, cat no 610051, 1:500 for IF); 6xHIS tag (Quiagen, 1014992, 1:2000 for WB and 1:1,000 for ELISA); talin-1 (BIO-RAD-Serotec, MCA725C, clone T1205, 1:2000 for WB, Sigma T3287, clone 8D4, 1:10,000 for WB); active integrin β1 (EMD Millipore MAB2247, clone 12G10, 1:100 for FACS, 1:500 for IF); integrin β1 (BD Pharmingen cat no. 552828), AIIB2 (Developmental Studies Hybridoma bank, 1:100 FACS) BCR (Cell signaling #3902, WB 1:1,000) Rac1/2/3 (Cell Signaling #2465, 1:1,000 for WB); actin (Santa Cruz, SC-1615, 1:2000-10000 for WB). HRP-conjugated secondary antibodies were from Santa Cruz (goat anti-rabbit SC-2004, goat anti-mouse SC-2031) or Jackson Immunoresearch (bovine anti-goat 805-035-180) and were used 1:5,000 for WB. IRDye-conjugated secondary antibodies were from LICOR. Alexa Fluor 488-conjugated anti-mouse IgG (715-545-150, 1:600 for IF); CyTM3-conjugated anti-Rabbit IgG (711-165-152, 1:600 for IF); R-Phycoerythrin-conjugated anti-mouse IgG (115-115-146, 1:100 for FACS) and Alexa Fluor 647-conjugated anti-goat IgG (805-605-180, 1:100 for FACS) were from Jackson Immunoresearch.

### Cells

Human embryonic kidney (HEK) 293 cells were maintained in DMEM supplemented with penicillin/streptomycin and 10% fetal bovine serum (FBS). Sf9 cells were grown at 27°C without CO_2_ in Grace Insect Medium supplemented with gentamicin and 10% FBS as monolayer. For protein expression Sf9 cells were grown in suspension in Sf-900 III serum-free medium (Life Technologies). HEK-DDR1b, HEK-Vector and HEK-DDR1b-Turbo were previously described (Chiusa et al., 2019; Jeffries et al., 2020; Borza et al., 2021). HEK-DDR1a, HEK-DDR1b-Y513A and HEK-DDR1a-turbo were generated as described (Jeffries et al., 2020; Borza et al., 2021). Briefly, cells were transfected with 1–2 µg of corresponding plasmid using Lipofectamine 2000 (Life Technologies, 11668027) and stable clones were isolated under puromycin (Sigma, P8833) or G418 (RPI, G640005) selection. To generate stably transfected HEK-Vector-GFP, HEK-DDR1a-GFP, HEK-DDR1b-GFP, HEK-DDR1a-Talin-GFP, HEK-DDR1b-Talin-GFP, and HEK-DDR1b-Y513A-Talin-GFP cells expressing Vector, DDR1a, DDR1b or DDR1b-Y513A were further transfected with the indicated GFP-constructs and stable clones were isolated under G418 selection. For transient transfections, 3 × 10^5^ HEK-DDR1b cells in 6 well plates were transfected with 2 µg of empty vector or BCR using Lipofectamine 2000. 24 h post transfection cells were serum starved for additional 24 h then collected for cell migration assay or Rac activation assay and for western blot analysis to verify overexpression.

### Flow Cytometry

To sort cells for equal DDR1 surface expression or GFP expression, cells were collected with trypsin and recovered in 10% FBS media. 2–3x10^6^ cells were incubated with antibodies to the extracellular domain of DDR1 (mAb 7A9 or AF2396) at 4°C for 1 h followed by incubation with either PE-conjugated anti mouse or Alexa-647-conjugated anti-goat secondary antibodies and sorted for equal DDR1 or DDR1 and GFP expression using a FACSAriall sorter from BD Biosciences, in the Research Flow Cytometry Core Laboratory at the Nashville VA Medical Center. Integrin β1 levels cells were determined by flow cytometry analysis as described (Borza et al., 2010). Briefly cells were incubated with AIIB2, washed then incubated with anti-rat-Alexa 488-conjugated secondary. Data were collected on a 3-laser BD LSR Fortessa (BD Biosciences) in the Vanderbilt Flow Cytometry Shared Resource (FCSR) and results were analyzed using FlowJo software.

### Cell Adhesion and Migration Assay

Cell adhesion and migration were performed as previously described (Borza et al., 2006; Abair et al., 2008). For cell migration, collagen I (Corning cat no. 54236) at 20 µg/ml or DC1 and DC1-GVMGFP at 30 µg/ml were added to transwells with 8-μm pores (Corning) in 20 mM acetic acid, overnight at 4°C. We used monomeric collagen I rather than collagen fibrils as monomeric collagen is commercially available, and this is the collagen described to study DDR1 activation and DDR1-mediated effects (Wang et al., 2006; Xu et al., 2012). Nonspecific binding sites were blocked with 1% bovine serum albumin (BSA) at 37°C for 1 h. Cells were collected with accutase (STEM Cell Technologies, cat. no 07920) washed and suspended in serum free DMEM supplemented with 1% BSA and 2 mM MgCl_2_. 2–5x10^4^ cells in 150 µl were added to the top of the transwell and cells and allowed to migrate for 4 h at 37°C for collagen I or 6 h at 33°C for DC1 and DC1-GVMGFP. Cells on the bottom of the transwell were fixed with 4% paraformaldehyde in PBS and stained with 2% crystal violet and migrated cells were counted on 4–8 randomly chosen fields.

For cell adhesion, collagen I, DC1 and DC1-GVMGFP (3–30 µg/ml) were added to 96 well plates (Thermo Scientific) in 20 mM acetic acid or phosphate buffer saline (PBS), overnight at 4°C. Nonspecific binding sites were blocked as indicated above. 5 × 10^4^ cells in 100 μl were added to each well and incubated at 37°C for 45 min for collagen I, or 33°C for 1 h for DC1 and DC1-GVMGFP. Non-adherent cells were removed by washing with PBS with Ca^2+^and Mg^2+^ and the attached cells were fixed with 4% paraformaldehyde, stained with 0.1% crystal violet (Sigma, 548-62-9) and lysed with 10% acetic acid and cell adhesion was quantified by reading the plates at 595 nm with a microtiter plate reader.

### Depletion of Integrin β1 and BCR by siRNA

Silencer Select Validated small interfering RNAs (siRNA) for integrin β1 sense 5′-GCA​GUU​GGU​UUU​GCG​AUU​Att and Silencer Select siRNA for BCR sense 5′-CAG​AAG​AAG​UGU​UUC​AGA​Att were obtained from Life Technologies. For depletion experiments, HEK-Vector, HEK-DDR1a or HEK-DDR1b were plated in 6 well plates at 3 × 10^5^ cells/well and transfected with 50 nM of each siRNA using Lipofectamine 2000 following the manufacturer protocol. After 24 h, the cells were re-transfected with the siRNA and after additional 48 h, they were collected and used for cell migration or Rac activation assays. FACS was used to ensure integrin β1 depletion while Western blot analysis was used to ensure downregulation of BCR.

### Western Blots

Proteins from the various cells were extracted using Cell Signaling Cell Lysis Buffer (#9803) following manufacturer instructions and protein concentration was determined using a BCA assay (Thermo Scientific). Equal amounts of total proteins were separated onto SDS-PAGE and subsequently transferred to nitrocellulose membranes. Membranes were blocked with either 5% BSA for detection of phosphorylated proteins or 5% milk and then incubated with various primary antibodies followed by the appropriate HRP or IRdye-conjugated secondary antibodies. Immunoreactive bands were identified using enhanced chemiluminescence (Perkin-Elmer, NEL 104001) according to the manufacturer’s instructions or the Odyssey CLx imaging system. Bands were quantified either by densitometry analysis using VisionWorksLS or using the Odyssey CLx software.

### Immunofluorescence

To visualize DDR1 in FAs, cells were plated in serum free medium on slides coated with collagen I (20 µg/ml) or fibronectin (10 µg/ml, Sigma). After 1 h the cells were fixed, in 4% paraformaldehyde in PBS for 15 min and then incubated with 5% BSA containing 0.3% Triton X-100 in PBS. After 2 h, cells were incubated with anti-DDR1 and anti-12G10 antibodies, anti-DDR1 antibody and FITC-phalloidin, or anti-DDR1 and anti-paxillin antibodies followed by the appropriate Cy3-or Alexa488-conjugated secondary antibodies. Cells were then analyzed under an epifluorescence microscope (Nikon) or a Nikon TiE microscope equipped with a Total Internal Reflection Fluorescence (TIRF) illuminator and 100x/1.49 objective.

### Recombinant Protein Expression and Purification

DDR1b cytoplasmic domains was previously described (Jeffries et al., 2020), DDR1a cytoplasmic domain was expressed using Bac-to-Bac baculovirus expression system following the manufacturer instructions, as described (Borza et al., 2017; Jeffries et al., 2020). Recombinant proteins were expressed in Sf9 cells, as described (Borza et al., 2017).

pET30a-Talin-HIS cDNA was transformed in competent ArcticExpress (DE3) *E. Coli*. For recombinant protein expression bacterial cells were grown at 30°C then induced with 1 mM isopropyl β-d-thiogalactoside (IPTG) (Sigma) 10°C for 20 h, pelleted, lysed with lysis buffer (Hepes 50 mM pH 6.8, NaCl 300mM, glycerol 10%, Tween 0.2%, DTT 0.5 mM) supplemented with lysonase (Novagen) and bacterial protease inhibitors (Sigma), clarified by centrifugation and then purified on a TALON metal affinity resin (Clontech) for the HIS-tagged proteins following manufacturer’s instructions To remove imidazole used for elution, proteins were applied on a Zeba Spin desalting columns then proteins were aliquoted and stored at −80°C.

DC1 and DC1-GVMGFP proteins were expressed in *E. coli* BL21 (Novagen), and purification was carried out by affinity chromatography on a HisTrap HP column (GE Healthcare) and subsequent dialysis against 20 mM acetic acid (regenerated cellulose, MWCO = 12–14 kDa). Protein purity was assessed by sodium dodecyl sulfate polyacrylamide gel electrophoresis (SDS-PAGE) followed by Coomassie blue staining. Protein concentrations were measured using the BCA protein assay (Pierce). Samples were stored at −80°C.

### Circular Dichroism Spectroscopy

Circular dichroism (CD) spectra were obtained on protein samples in phosphate buffered saline (PBS) using a Jasco J720 spectropolarimeter in a thermostatically-controlled cuvette (0.5 mm path length). Data were collected over 250 to 190 nm wavelengths (integration = 1 s at 0.2 nm intervals; bandwidth = 1 nm). Ten scans were collected for each sample, and the buffer contribution was subtracted. The ellipticity at 220 nm was monitored from 25 to 45°C with an average temperature slope of 10°C/h to assess thermal transitions.

### ELISA

ELISA assays were performed as previously described (Olaru et al., 2014). Briefly, 96 well plates (Nunc, maxisorp) were coated with DDR1a or DDR1b cytoplasmic tails in 50 mM sodium bicarbonate buffer pH 9.5 overnight at 4°C at the indicated concentrations, blocked with 0.5% bovine serum albumin (BSA) protease and IgG-free (Sigma, cat. no A3059) in 50 mM Tris, pH 7.5, and 150 mM NaCl and sequentially incubated with anti-DDR1 (Santa Cruz) or biotinylated anti-HIS, followed by HRP-conjugated anti-rabbit or HRP-conjugated avidin (Life Technologies). Finally HRP substrate (Bio-Rad, cat no 172–1,064) was added to the wells and the absorbance was measured at 650 nm.

### Solid Phase Binding Assay

For DDR1 binding assays purified recombinant talin head domain was coated on 96 well plates (Nunc, maxisorp) and plates were blocked as described above. DDR1 cytoplasmic constructs were added at increasing concentrations in 50 mM Tris, pH 7.5, 150 mM NaCl, 0.1% BSA, 2 mM DTT and 0.1% Tween 20 and plates were sealed and incubated overnight at 4°C. The plates were then washed with 50 mM Tris, pH 7.5, 150 mM NaCl, 0.1% Tween and bound DDR1 was detected with anti-DDR1 antibody (Santa-Cruz) followed by HRP-conjugated anti-Rabbit secondary. As control for nonspecific binding, BSA-coated wells and THD FERM coated wells were incubated with anti-DDR1 antibody.

### Streptavidin Pull-Down

Serum starved HEK cells expressing DDR1a-turbo, DDR1b-turbo or the biotin ligase turbo alone were plated on collagen I (30 µg/ml in 20 mM acetic acid) for 1–3 h in the presence of biotin (50 µM). Cells were washed with PBS then lysed in 50 mM Tris, pH 7.5, 150 mM NaCl, 1% IGEPAL, 0.4% SDS, 1 mM EGTA supplemented with protease and phosphatase inhibitors then biotinylated proteins were isolated using streptavidin-agarose (Thermo Scientific) following the manufacturer instructions. Proteins were eluted by denaturation in sample buffer at 95°C for 5 min then analyzed by western blot analysis. Input lysates shown on the figure represents 2–5% of the lysate used for the streptavidin pull down.

### G-Lisa Activation Assay

Rac1-GTP levels were measured using the colorimetric G-LISA kit (Cytoskeleton #BK128) following the manufacturer’s instructions. Briefly, 24 h serum starved cells were trypsinized and either kept in suspension or plated on collagen I (30 µg/ml) at 37°C. After 30 min, they were washed with cold PBS then lysed using G-Lisa lysis buffer and equal amounts of proteins were added to plates coated with a Rac-GTP-binding protein. Bound active Rac, was detected with a Rac-specific antibody followed by an HRP-conjugated secondary antibody. The activated Rac1-GTP values were normalized for total Rac1 levels that were assessed by Western blot analysis.

### *In Vivo* Lung Colonization

The *in vivo* experiments were approved by the Vanderbilt’s Institutional Animal Care and Use Committee and performed according to institutional animal care guidelines. Mice were housed in an AALAC-accredited animal facility following NIH guidelines. HEK-Vector-GFP, HEK-DDR1a-GFP, or HEK-DDR1b-GFP were injected via the tail vein into athymic nude male mice, 12–13 weeks old (*n* = 10 for HEK-Vector and HEK-DDR1b and 12 for HEK-DDR1a, 2 × 10^5^ cell/animal in 200 µl). Two weeks after the injection, the mice were sacrificed, the lungs were removed and the numbers of GFP positive colonies within the lung parenchyma were evaluated by placing the lungs under an epifluorescence microscope and counting all the GFP-positive tumors in the lung. 2–3 images/animal were taken to document presence or absence of tumors in the lung.

### Macromolecular Modelling of DDR1b-Talin Complex

Currently, there are no reported structures of DDR1b juxtamembrane region deposited in Protein Data Bank (PDB), nor are there any structures which could serve as templates for homology modeling. Hence we devised a protocol that combines comparative modeling with *de novo* structure prediction using the Rosetta software for protein modeling. In order to produce a tentative model of DDR1b interaction with FERM domain, we have relied on a structure of galline talin F3 domain in complex with a chimeric murine β3 integrin-PIP kinase peptide (PDB ID: 2h7e (Wegener et al., 2007)). As the integrin W739 residue is highly evolutionarily conserved and presumably equivalent to residue W534 of DDR1b, we have threaded the sequence of DDR1b linker (A505-Q541 encompassing an NPxY motif, Figure 3A) onto the integrin structure, enforcing the same initial conformation. The resultant starting structure of the DDR1b peptide in complex with talin has then been subject to *FlexPepDock ab-initio*, a Rosetta-based protocol for simultaneous folding, docking and refinement of peptides onto their protein interaction partners (Raveh et al., 2011). Docking and refinement of DDR1 has been performed under three regimes: 1) relaxation and repacking, 2) fully flexible docking, relaxation and repacking and 3) analogous to (ii), but with enforced anchoring between W359 in talin and W534 in DDR1b. For each regime we have conducted 200,000 independent energy minimization trajectories. The resultant models were analyzed based on the interface energy score *dG*_*separate* (Lewis and Kuhlman, 2011), that is a difference in computed energy of a complex and the energy of both chains pulled apart and minimized. The best scoring poses in all three regimes were very similar and captured the same conformation of DDR1b-talin interaction (Figure 3A).

### Computational Mutagenesis Evaluation of Validity of Proposed Pose

In order to evaluate the robustness and estimate the energetics of interaction, we have conducted an extensive computational mutagenesis experiment. For each amino acid position indicated by Rosetta as contributing significantly to DDR1b/talin binding, we mutated it to each of the 19 other genetically encoded amino acids. The mutated starting structures have then been relaxed, rendering 50 models for each residue type. Each of the models has been subject to binding energy calculations by means of Rosetta InterfaceAnalyzer (Lewis and Kuhlman, 2011) and the most favorable pose in terms of computed binding energy across interface (dG_cross) has been used for subsequent computation. The binding energy of a mutated pose has been subtracted from the binding energy of the “native” pose, allowing us to roughly quantify the effects of mutations. Full results of the computational mutagenesis can be found in Supplementary Figure S4.

### Image Analysis

Images were captured with a Nikon epifluorescence microscope using a 40x or a 60x oil immersion objective using the appropriate fluorescence channels or Nikon TiE microscope equipped with a Total Internal Reflection Fluorescence (TIRF) illuminator and 100x/1.49 objective. The individual images were converted to tiff files and then Adobe Photoshop was used to produce the final figures. Mean fluorescence intensity was quantified using ImageJ (National Institutes of Health, Bethesda) and the JaCoP plugin Manders’ overlap coefficient (Bolte and Cordelieres, 2006) was used to determine the degree of overlapping between green and red signals. 10–13 cells were analyzed with 3–4 focal adhesions analyzed per each cells. Data points represent single FA values.

### Statistical Analysis

Data are shown as mean ± SEM. Statistical analysis was performed using GraphPad Prism software (9 version). To evaluate the statistical significance of the differences between groups we used unpaired two tailed *t-test* for two groups or One-way ANOVA (assuming Gaussian distribution) followed by Tukey’s test for pairwise comparisons within multiple groups A *p* ≤ 0.05 was considered to be statistically significant.

## Data Availability

The raw data supporting the conclusion of this article will be made available by the authors, without undue reservation.
