# Supplementary figures and images for: The Collagen Receptor Discoidin Domain Receptor 1b Enhances Integrin β1-Mediated Cell Migration by Interacting With Talin and Promoting Rac1 Activation
Source: Front Cell Dev Biol. 2022 Mar 3;10:836797. doi: 10.3389/fcell.2022.836797 (PMC8928223; doi:10.3389/fcell.2022.836797)

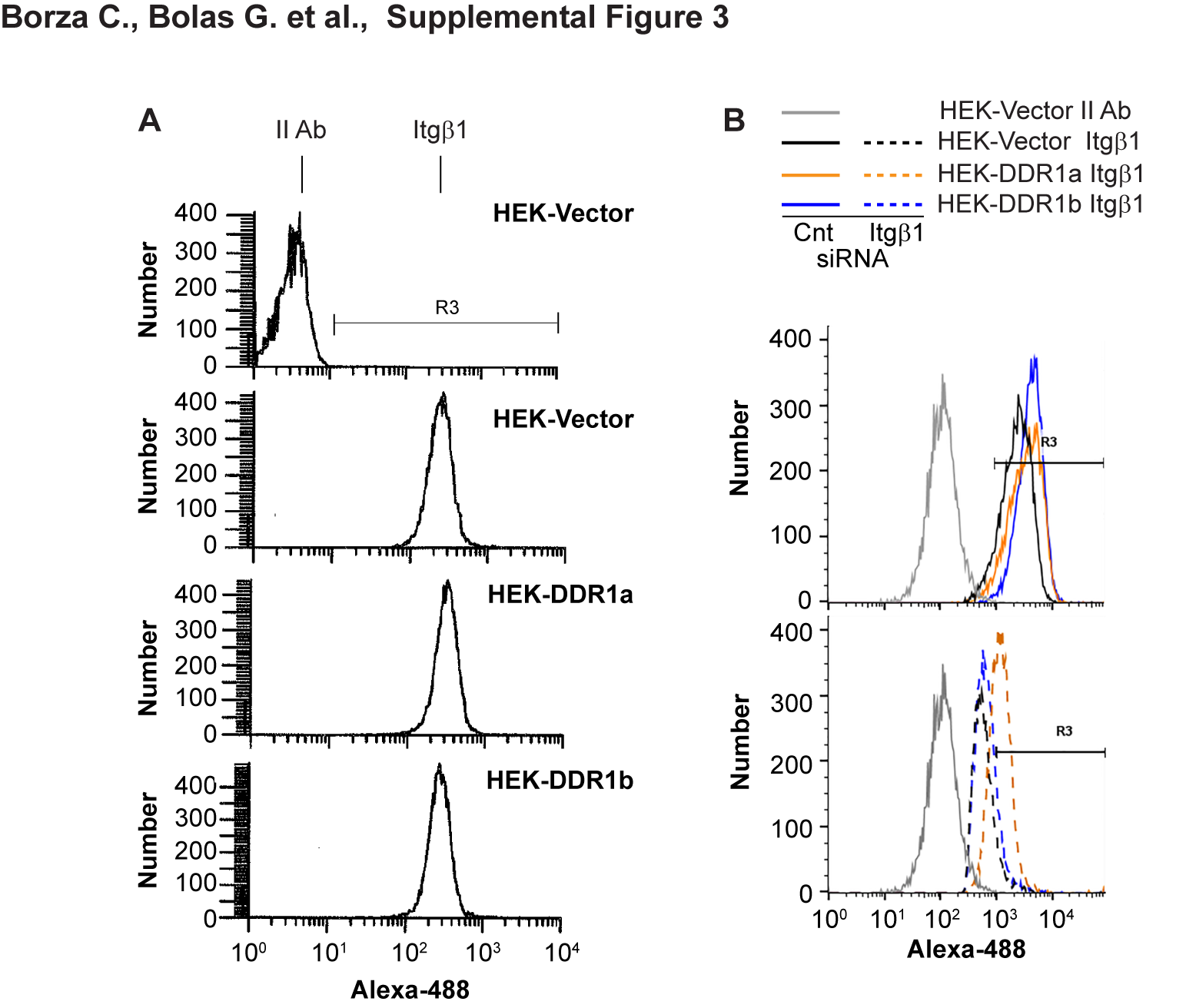

Supplement: Supplementary file 1 [file Image3.TIF]

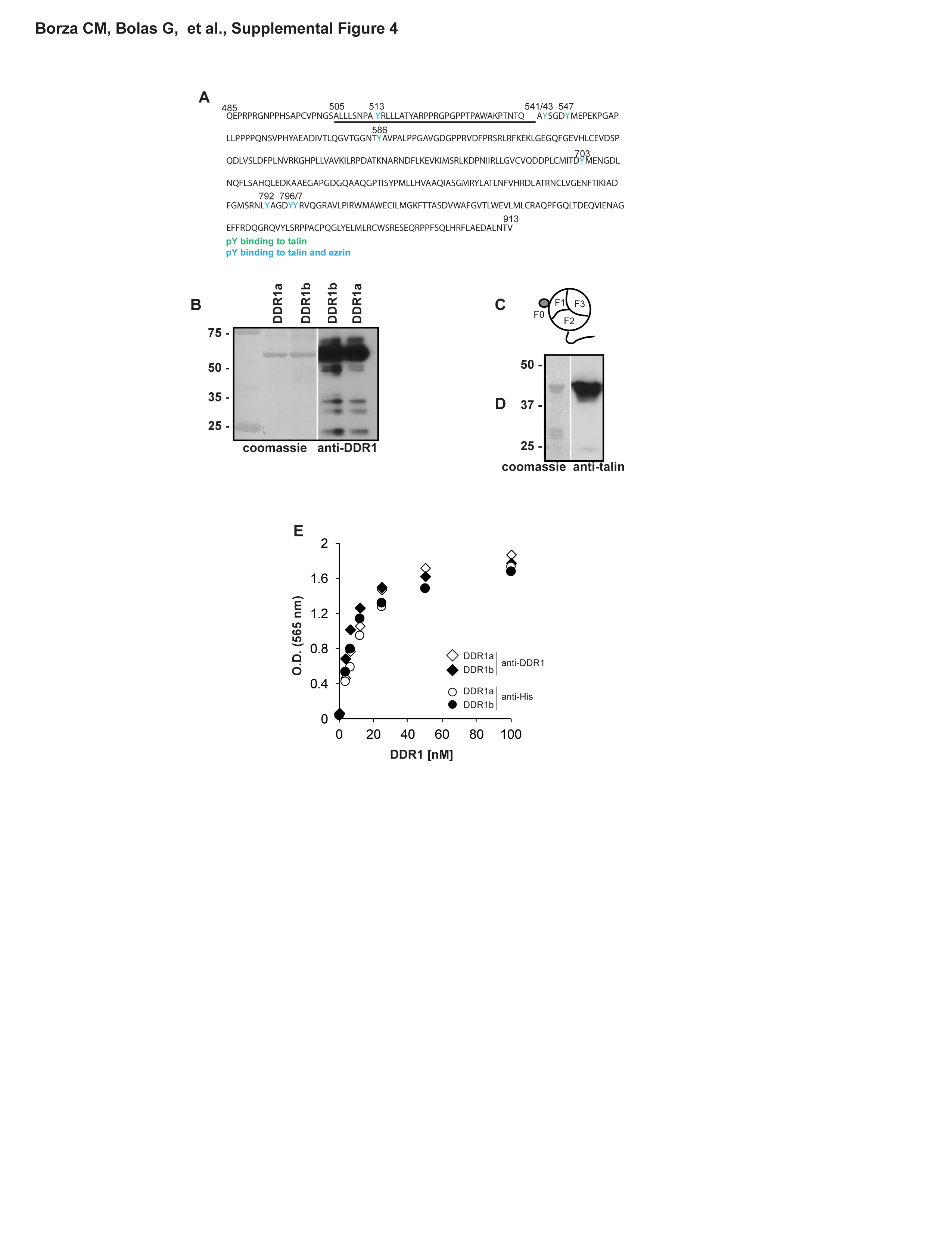

Supplement: Supplementary file 2 [file Image4.TIF]

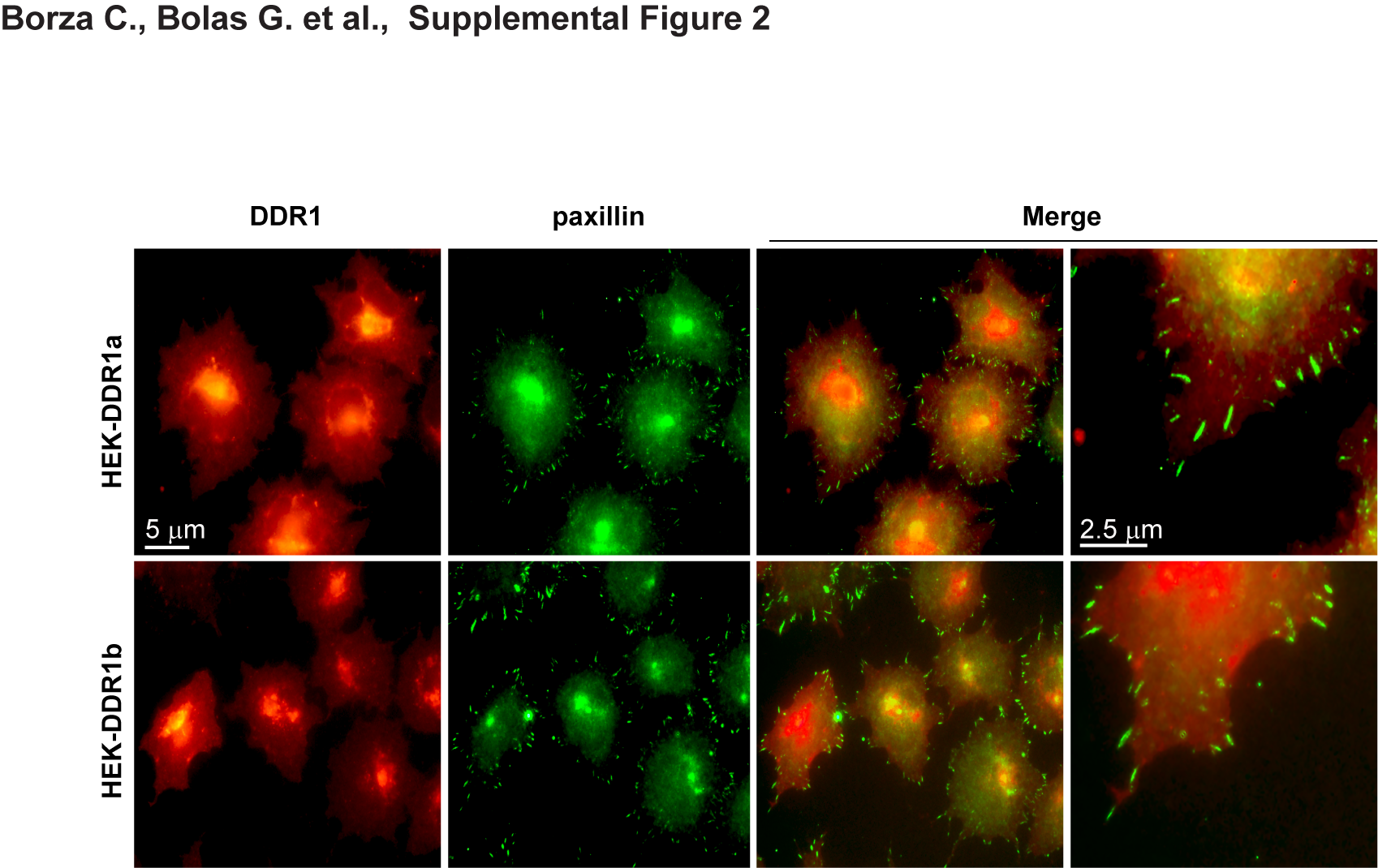

Supplement: Supplementary file 3 [file Image2.TIF]

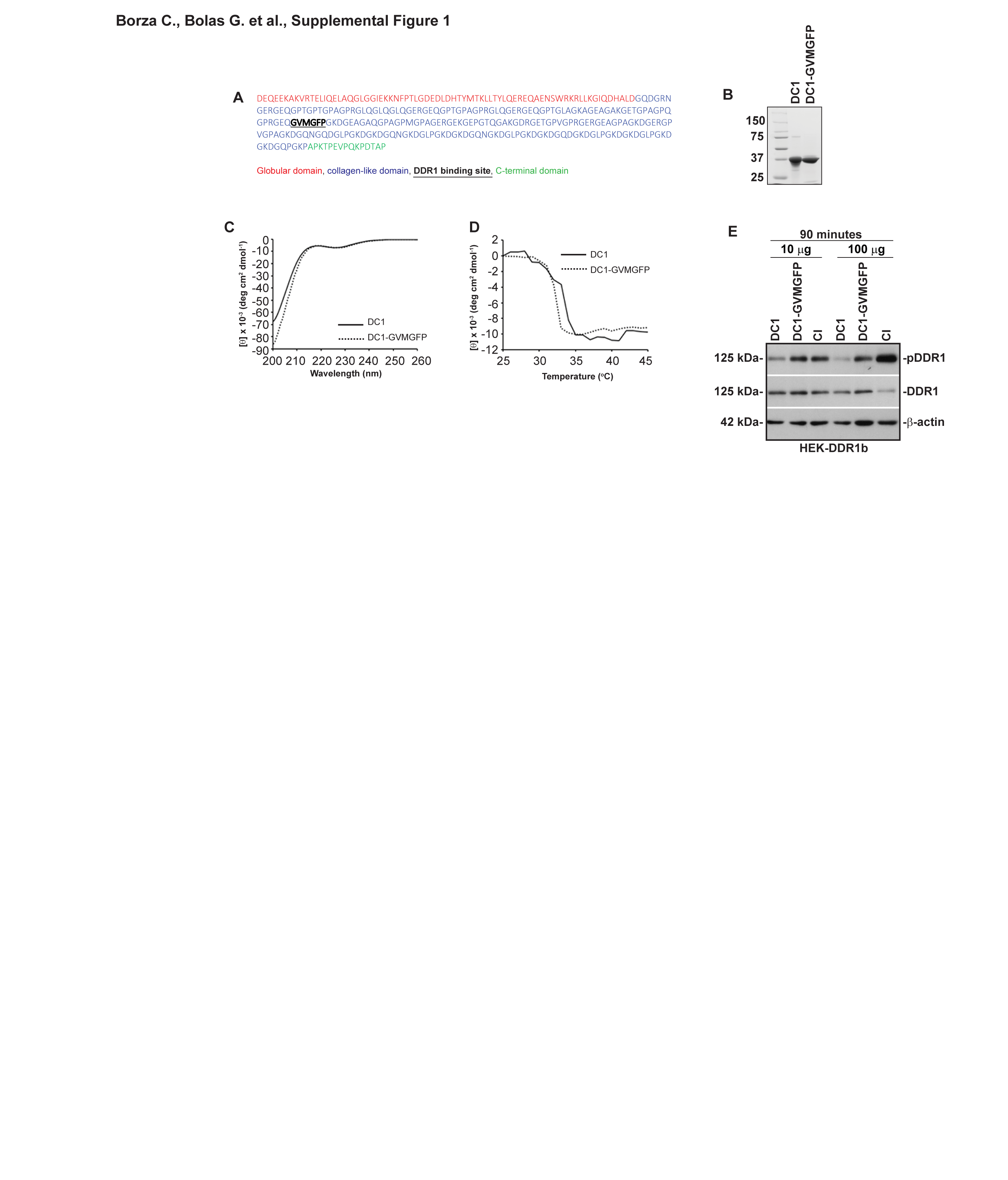

Supplement: Supplementary file 4 [file Image1.TIF]

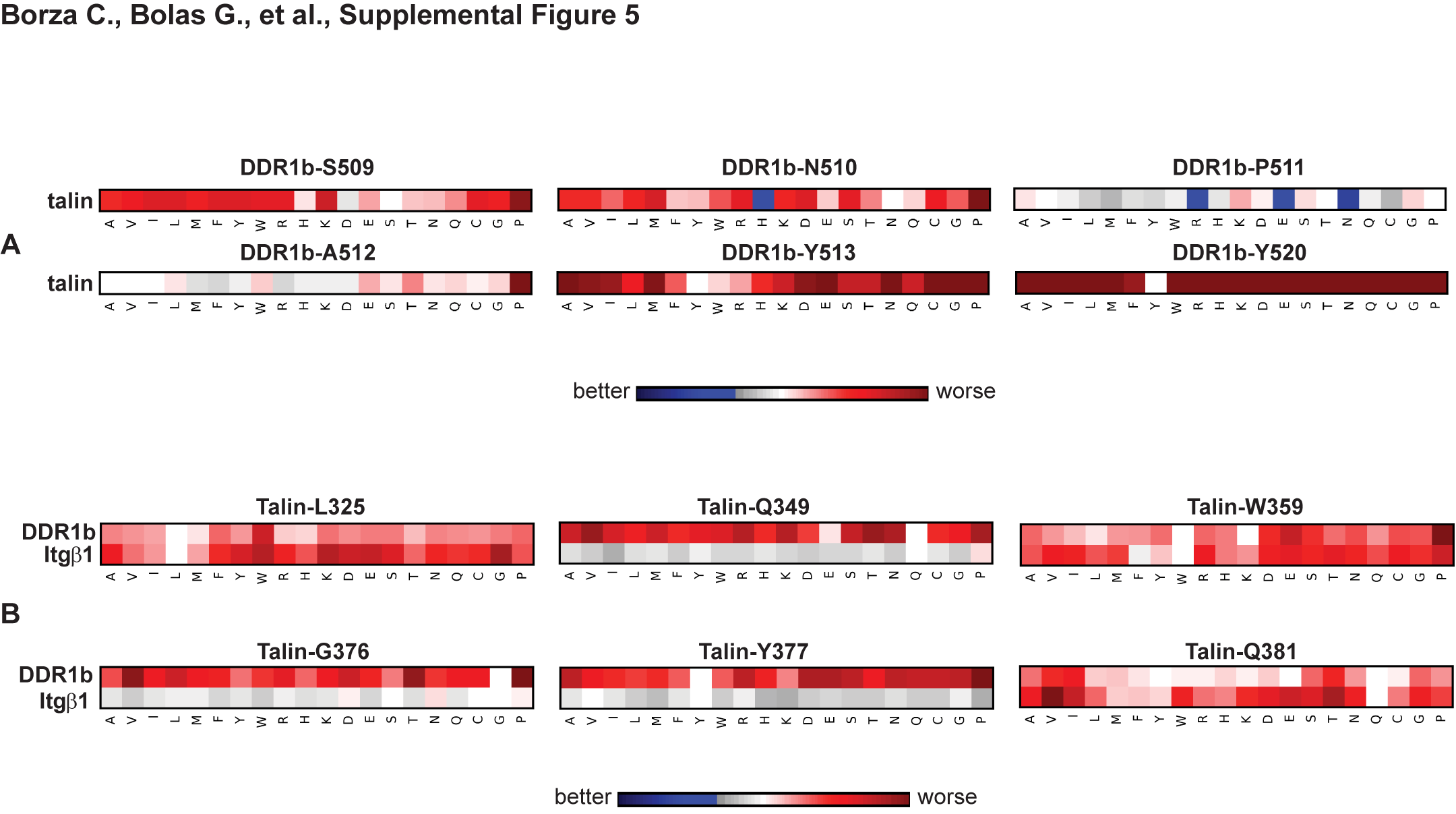

Supplement: Supplementary file 6 [file Image5.TIF]
