# Supplementary material for: The Collagen Receptor Discoidin Domain Receptor 1b Enhances Integrin β1-Mediated Cell Migration by Interacting With Talin and Promoting Rac1 Activation
Source: Front Cell Dev Biol. 2022 Mar 3;10:836797. doi: 10.3389/fcell.2022.836797 (PMC8928223; doi:10.3389/fcell.2022.836797)
Supplement: Supplementary file 5 [file DataSheet1.pdf]

Supplementary Material

Supplemental Figure 1

Purification and analysis of DDR-selective substrata. (**A**) The DDR binding site was inserted into the collagen-like region of DC1 via overlap extension PCR. (**B**) Coomassie staining showing purity of DC1 and DC1-GVMGFP proteins (5 µg each). (**C**) CD spectra of DC1 and DC1-GVMGFP with peak at 220 nm that is indicative of triple helical structures. (**D**) Thermal transitions of DC1 and DC1-GVMGFP monitored at 220 nm to demonstrate a melting temperature of ~33°C. (**E**) Serum-starved HEK-DDR1b were treated with the indicated amounts of DC1, DC1-GVMGFP or collagen I for 90 minutes and the levels of phosphorylated and total DDR1 were analyzed by Western blot.

Supplemental Figure 2

DDR1b does not localize to focal adhesions in cells plated on fibronectin. Images of HEK-DDR1a and HEK-DDR1b cells plated on fibronectin (10 µg/ml) for 1 hour and then costained with anti-DDR1 (red) and anti-paxillin (green) antibodies.

Supplemental Figure 3

Integrin β1 expression in HEK-Vector, HEK-DDR1a and HEK-DDR1b cells. Flow cytometry analysis of integrin β1 levels in Vector- DDR1a-, or DDR1b-expressing HEK cells untransfected (**A**) or transfected (**B**) with Cnt (continuous line) or Itgβ1 (dotted line) siRNA. One representative experiment of 3 independent experiments is shown.

Supplemental Figure 4

Purification of DDR1a and DDR1b cytoplasmic domains. (**A**) Sequence of DDR1b cytoplasmic domain (a.a. 485-913) showing the unique 37 amino acids containing an NPxY motif (bold and underlined) and putative talin and/or ezrin binding tyrosine residues described in (Lemeer et al.,

2012). (**B**) Coomassie stain of purified His-tagged DDR1a, DDR1b cytoplasmic domains (2 µg each) and western blot showing the anti-DDR1 antibody recognizes the recombinant proteins (20 ng each). (**C**) Schematic representation of the talin 1 construct (THD, a.a. 1-433) used for the binding assay (**D**) Coomassie stain of purified THD (2 µg) and Western blot showing recombinant protein is recognized by anti-Talin antibody (20 ng). (**E**) ELISA assay of Immobilized His-tagged DDR1a and DDR1b cytoplasmic domains (0-100 ng/ml) incubated with anti-DDR1 or anti-His antibody to ensure that the antibodies equally recognize the two purified proteins. One representative experiment performed in triplicates is shown. Two independent experiments were performed with similar results.

Supplemental Figure 5

Importance of individual amino acids for talin-DDR1b binding. (**A**) Panels show heat map of influence of amino acid substitution on computed DDR1b (505-541)-talin F3 domain binding energy. Each of the 6 panels shows effects of mutating a particular amino acid of DDR1b to other side chains on binding to talin F3 domain. (**B**) Panels show heat map of influence of amino acid substitution on computed talin F3 domain and DDR1b and integrin β1 (Itgβ1) cytoplasmic tail (716-749) binding energy. Each of the 6 panels shows effects of mutating a particular amino acid of talin F3 domain to other side chains on binding to DDR1b or Integrin β1. Red squares indicate decreased/loss binding, white squares indicate no energetic effect, blue squares indicate improved binding. The results in the figure were obtained from the analysis of the most favorable binding pose for each of the substitutions.
